# Supplementary material for: Multi-modal locomotor costs favor smaller males in a sexually dimorphic leaf-mimicking insect
Source: BMC Ecol Evol. 2022 Mar 28;22:39. doi: 10.1186/s12862-022-01993-z (PMC8962604; doi:10.1186/s12862-022-01993-z)
Supplement: Supplementary file 1 — Additional file 1: Table S1. Scaling relationships between body size and various morphological measurements. Figure S1. Morphological measurements for females (left) and males (right) P. philippinicum. Figure S2. Scaling relationships between various morphological traits (body mass (A), body area (B), body circularity (C), body aspect ratio (D), antenna length (E), front femur length (F), total wing area (G), wing loading (H) and flight muscle dry mass (I)) and body length in males (blue) and females (red). Figure S3. Set-up for flight trials. Figure S4. Analysis of a male P. philippinicum flight trial. Figure S5. Mean horizontal velocity (A) and mean resultant velocity (B) as a function of wing loading. Figure S6. Instantaneous velocity at landing as a function of male body mass. Figure S7. Calculation of wing stroke amplitude. Figure S8. Acquisition of multiple 2D photographs from different angles of a male mounted on a pin to reconstruct a single 3D model using photogrammetry. Figure S9. Leaf insect model and CFD simulation results. [file 12862_2022_1993_MOESM1_ESM.pdf]

Supplementary Material

Multi-modal locomotor costs favor smaller males in a sexually dimorphic leaf-mimicking insect

Romain P. Boisseau[1,*], Thies H. Büscher[2], Lexi J. Klawitter[1], Stanislav N. Gorb[2], Douglas J. Emlen[1], Bret W. Tobalske[1]

[1] Division of Biological Sciences, University of Montana, 32 Campus Dr, Missoula, MT 59812, United States of America

[2] Functional Morphology and Biomechanics, Zoological Institute, Kiel University, Am Botanischen Garten 9, D-24098 Kiel, Germany

This file includes:

Tables S1

Figures S1-S9

Legends for Videos S1-S3

Table S1: Scaling relationships between body size and various morphological measurements. Results of type I ANOVA from linear models contrasting the differences between sexes in terms of scaling relationships between body size (body length) and body mass, area, circularity, aspect ratio, antenna length or front femur length (Fig. S2). The scaling relationships for male hindwing area, female forewing area, male wing loading and male flight muscle mass with body size are also presented. Scaling exponents β and the corresponding 95% confidence intervals are shown in comparison to isometric expectations. Significant effects (i.e., p <0.05) are bolded.

Response variable (log_{10} transformed)	Explanatory variables	F	df1	df2	P	Isometric slope	Slope β [95% CI]
Body mass	**log_{10}(body length)**	**3075**	**1**	**40**	**<0.001**	3	2.89 [1.61, 4.16]
	**sex**	**14.2**	**1**	**40**	**<0.001**		
	interaction	0.002	1	40	0.97		
Body area	**log_{10}(body length)**	**12897**	**1**	**40**	**<0.001**	2	1.82 [1.4, 2.24]
	**sex**	**58.8**	**1**	**40**	**<0.001**		
	interaction	1.56	1	40	0.22		
Body circularity	**sex**	**1675**	**1**	**40**	**<0.001**	0	0.11 [-0.27, 1.12]
	log_{10}(body length)	1.15	1	40	0.29		
	interaction	0.64	1	40	0.43		
Body aspect ratio	**sex**	**1968**	**1**	**40**	**<0.001**	0	0.36 [-0.09, 0.8]
	log_{10}(body length)	0.69	1	40	0.41		
	interaction	3.53	1	40	0.07		
Antenna length	**log_{10}(body length)**	**15396**	**1**	**30**	**<0.001**	1	1.22 [0.72, 1.72]
	**sex**	**670**	**1**	**30**	**<0.001**		
	interaction	0.01	1	30	0.92		
Front leg length	**log_{10}(body length)**	**1047**	**1**	**40**	**<0.001**	1	1.15 [0.7, 1.59]
	sex	1.06	1	40	0.31		
	interaction	0.32	1	40	0.57		
Male hindwing area	**log_{10}(body length)**	**23.8**	**1**	**23**	**<0.001**	2	1.86 [1.07, 2.65]
Female forewing area	**log_{10}(body length)**	**79.4**	**1**	**17**	**<0.001**	2	1.89 [1.44, 2.34]
Male wing loading	**log_{10}(body length)**	**5.23**	**1**	**23**	**0.03**	1	1.08 [0.1, 2.06]
Male flight muscle mass	**log_{10}(body length)**	**9.98**	**1**	**21**	**0.005**	3	5.2 [1.78, 8.65]

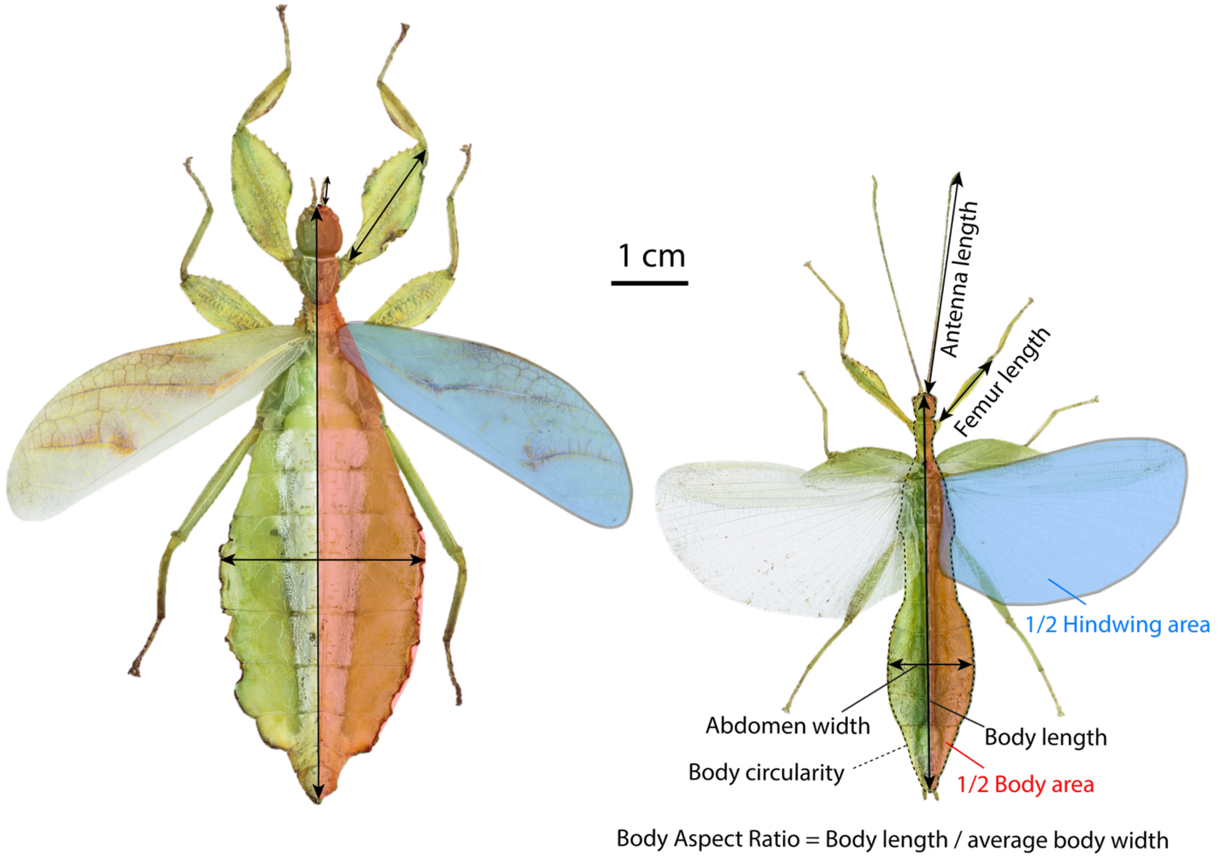

Figure S1: Morphological measurements for females (left) and males (right) *P. philippinicum*.

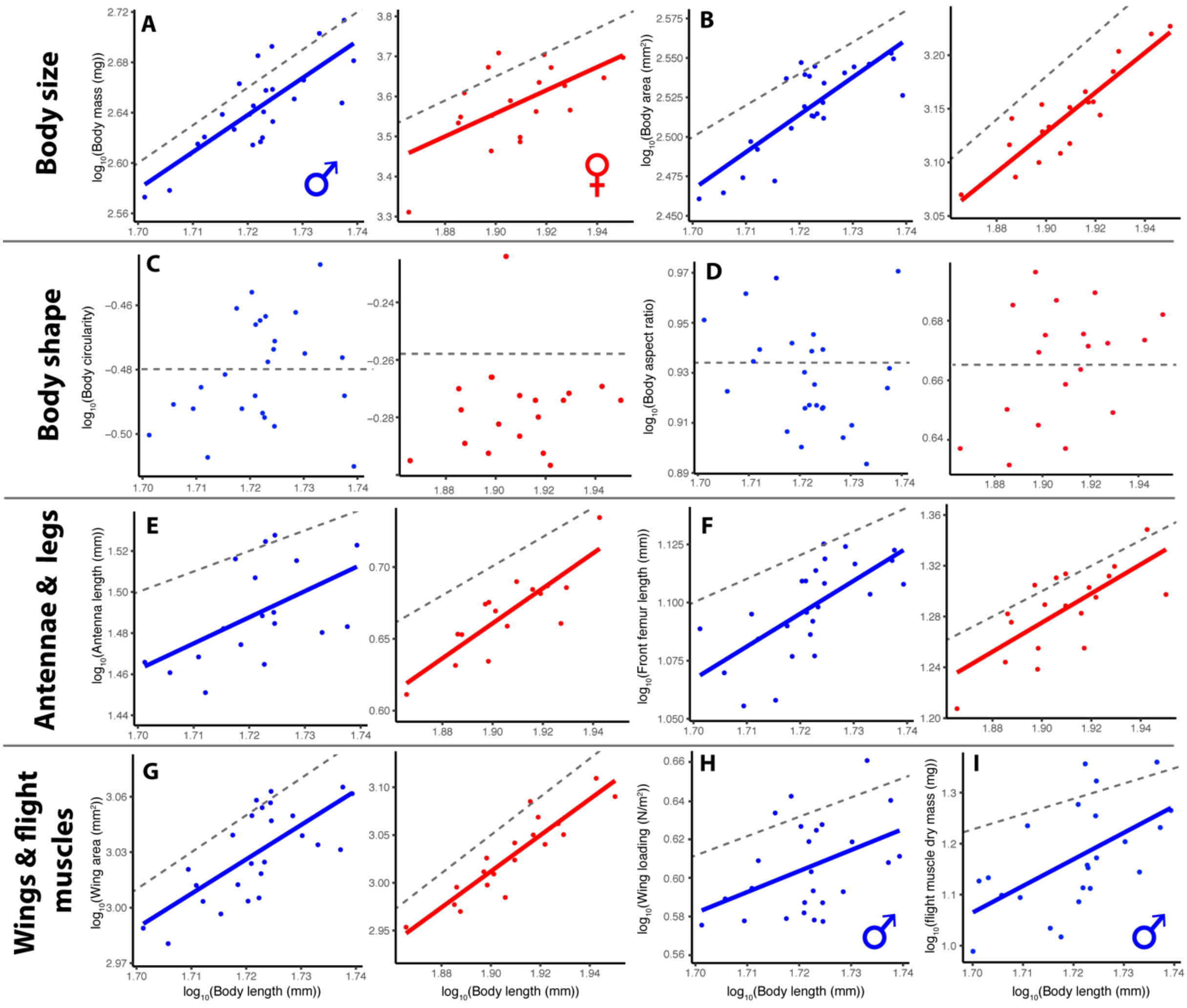

Figure S2: Scaling relationships between various morphological traits (body mass (**A**), body area (**B**), body circularity (**C**), body aspect ratio (**D**), antenna length (**E**), front femur length (**F**), total wing area (**G**), wing loading (**H**) and flight muscle dry mass (**I**)) and body length in males (blue) and females (red). Dashed lines show an isometric slope (arbitrary intercept). Wing area refers to hindwings for males, forewings for females. Wing loading and flight muscle mass are only shown for males as females are flightless.

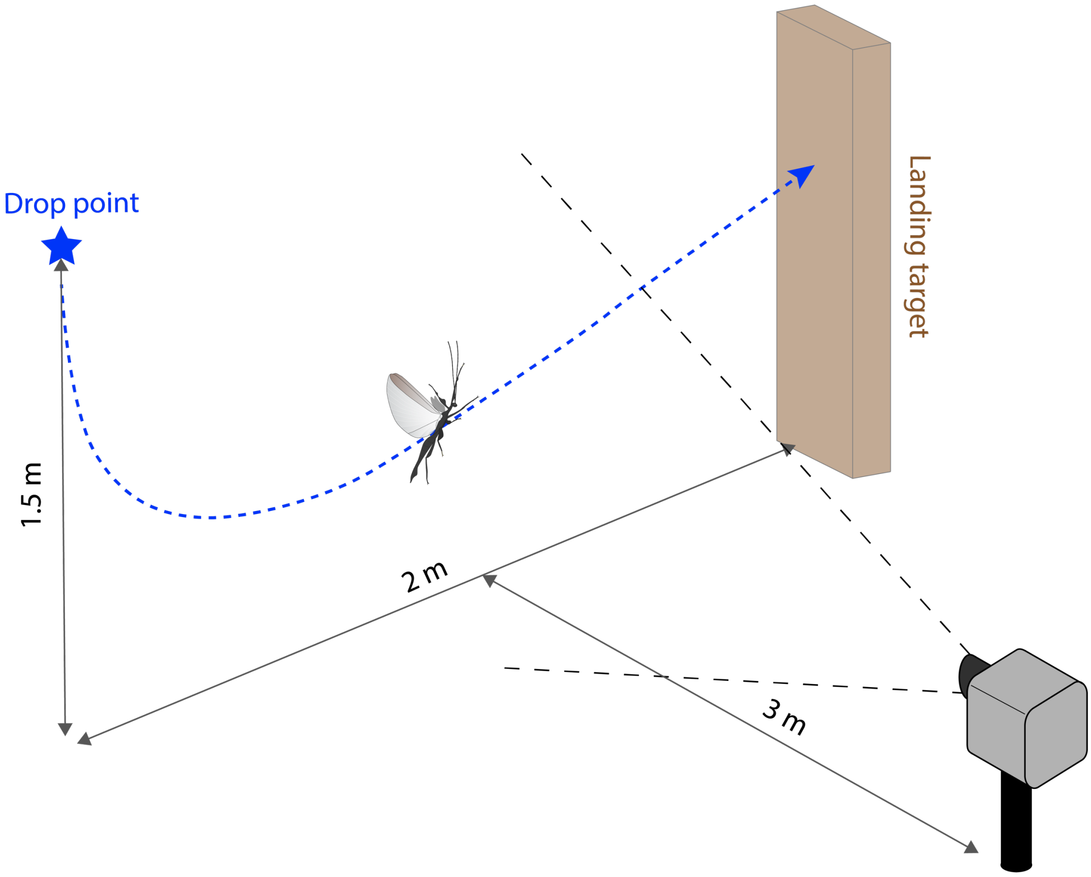

Figure S3: Set-up for flight trials.

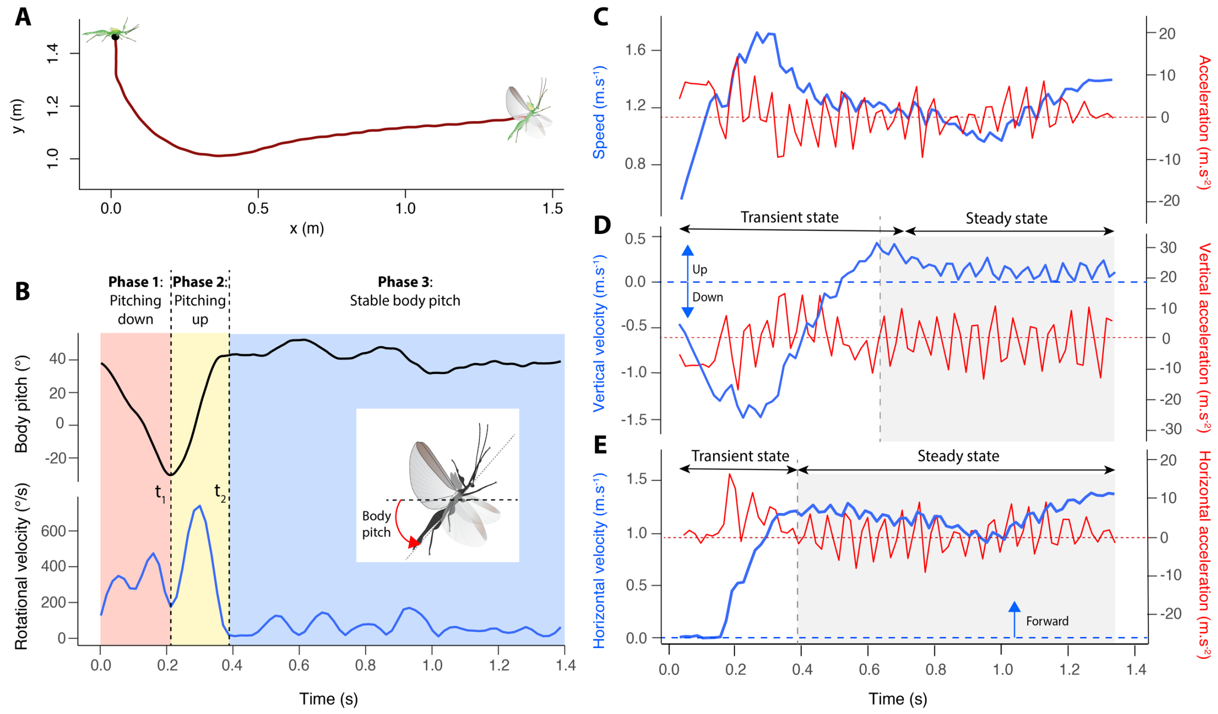

Figure S4: Analysis of a male *P. philippinicum* flight trial. **A**: Smoothed trajectory. Cartoons show the typical postures of the insect at the beginning and end of the trial. **B**: Body pitch and body pitch rotational velocity as a function of time. Colors indicate different phases during the trial defined by t_1 – i.e., to the time when body pitch reaches its absolute minimum – and t_2 – i.e., the time when body pitch stabilizes. **C**: Body speed and overall acceleration as a function of time. **D**: Vertical body velocity and acceleration as a function of time. Positive values indicate an upward velocity or acceleration. **E**: Horizontal body velocity and acceleration as a function of time. Positive values indicate a forward velocity or acceleration. In **D** and **E**, the boundary between the transient and steady state corresponds to the time when velocity (vertical or horizontal) stabilizes.

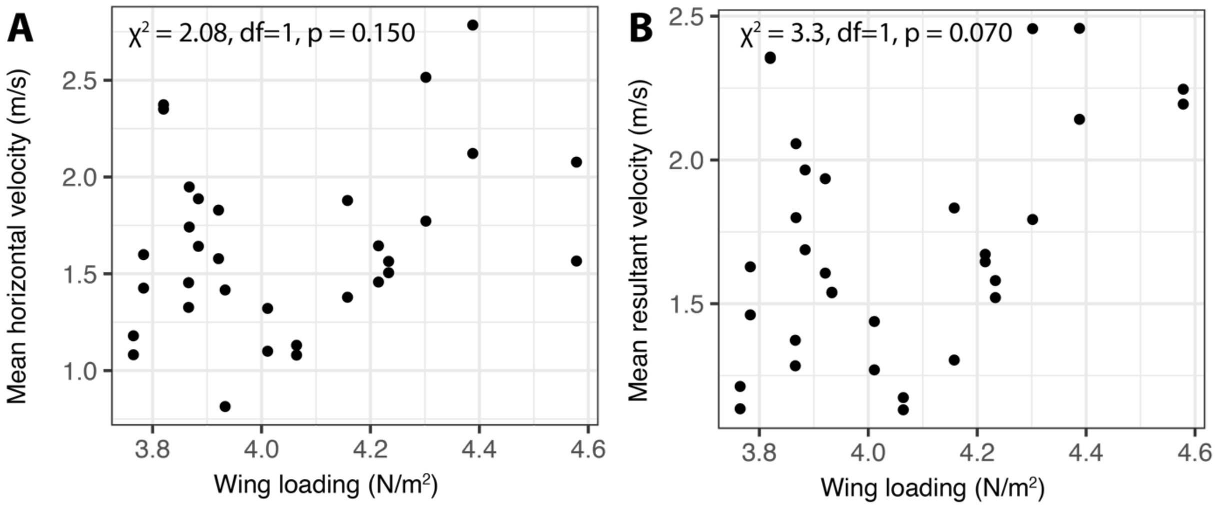

Figure S5: Mean horizontal velocity (**A**) and mean resultant velocity (**B**) as a function of wing loading. Mean horizontal velocity was calculated after reaching a steady state (Fig. S4E). Mean resultant velocity was calculated after stabilization of body angle (Fig. S4B). Each dot represents a trial (two per individual). Outputs from linear mixed effect models and corresponding likelihood ratio tests are shown on each graph.

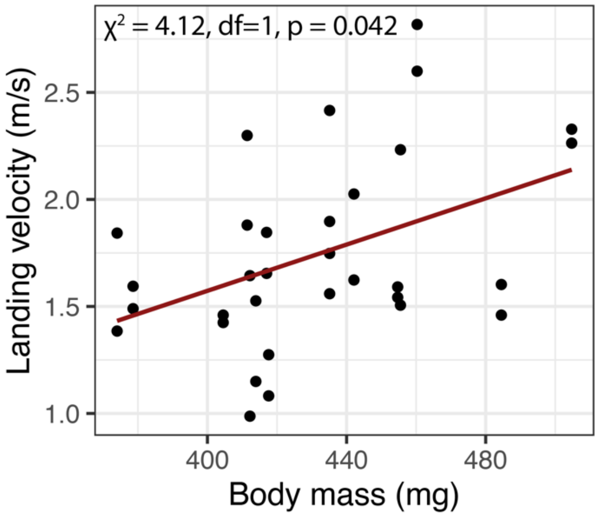

Figure S6: Instantaneous velocity at landing as a function of male body mass. Each dot represents a trial (two per individual). The output from a linear mixed effect model and the corresponding likelihood ratio test is shown on the graph (see methods).

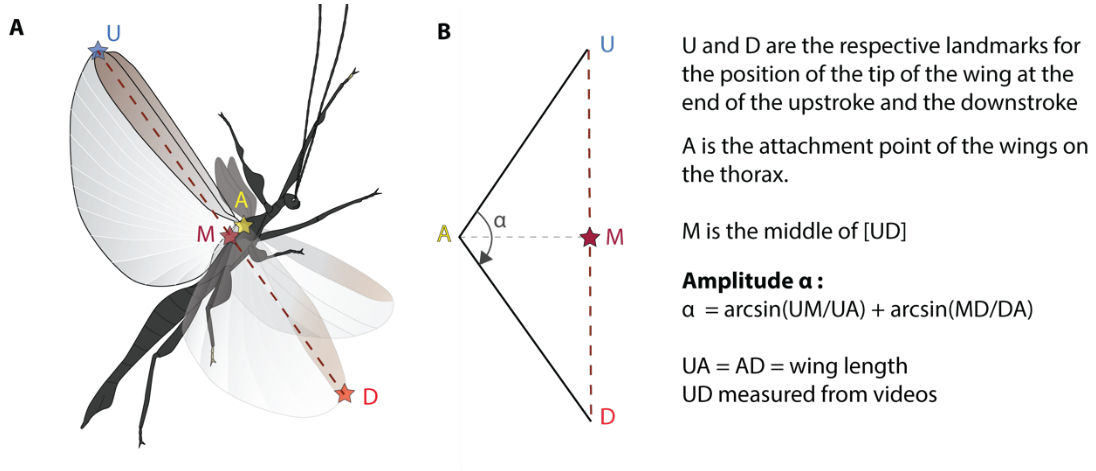

Figure S7: Calculation of wing stroke amplitude. **A:** side view of the animal with the relevant landmarks. **B:** landmarks in frontal view.

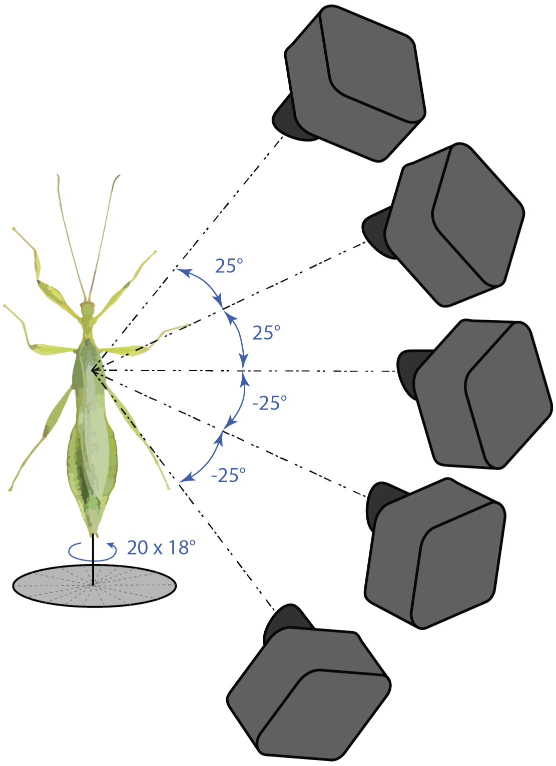

Figure S8: Acquisition of multiple 2D photographs from different angles of a male mounted on a pin to reconstruct a single 3D model using photogrammetry.

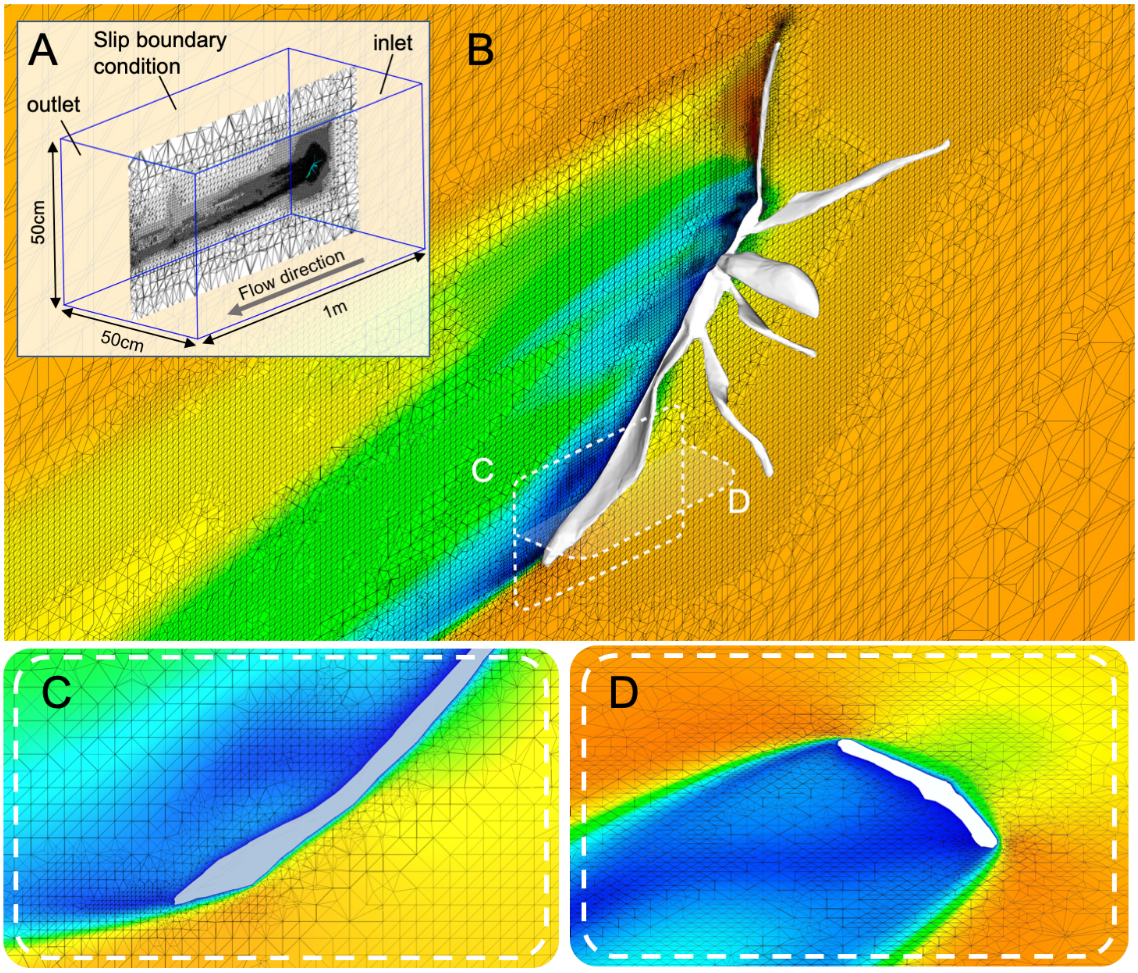

Figure S9: Leaf insect model and CFD simulation results. **A:** Meshed CFD domain. **B:** Detail of the mesh around the phasmid model along the median sagittal plane. **C:** Detail of the mesh around the tip of the abdomen along the median sagittal plane. **D:** Detail of the mesh around the middle of the abdomen along a cross-sectional plane. Colors in **B-D** represent air velocity (see scale in figure 6).

Video S1: Example of a flight trajectory from a relatively light male (374.0 mg)

Video S2: Example of a flight trajectory from a relatively heavy male (504.7mg)

Video S3: Detail of a male leaf insect ascending.
